# Supplementary material for: Modeling glioblastoma heterogeneity as a dynamic network of cell states
Source: Mol Syst Biol. 2021 Sep 16;17(9):e10105. doi: 10.15252/msb.202010105 (PMC8444284; doi:10.15252/msb.202010105)
Supplement: Supplementary file 6 — Source Data for Figure 5 [file MSB-17-e10105-s004.zip › Figure5A_sourcedata/GSEA_3071/hallmarks_stateV.GseaPreranked.1623742546329/HALLMARK_G2M_CHECKPOINT.html]

Details for gene set HALLMARK\_G2M\_CHECKPOINT[GSEA]

|  || Dataset | state43071 |
| Phenotype | NoPhenotypeAvailable |
| Upregulated in class | na\_pos |
| GeneSet | HALLMARK\_G2M\_CHECKPOINT |
| Enrichment Score (ES) | 0.6318644 |
| Normalized Enrichment Score (NES) | 3.658428 |
| Nominal p-value | 0.0 |
| FDR q-value | 0.0 |
| FWER p-Value | 0.0 |
Table: GSEA Results Summary

  

Fig 1: Enrichment plot: HALLMARK\_G2M\_CHECKPOINT      
 Profile of the Running ES Score & Positions of GeneSet Members on the Rank Ordered List

  

| PROBE | GENE SYMBOL | GENE\_TITLE | RANK IN GENE LIST | RANK METRIC SCORE | RUNNING ES | CORE ENRICHMENT || 1 | NUSAP1 |  |  | 6 | 2.001 | 0.0150 | Yes |
| 2 | TOP2A |  |  | 14 | 1.842 | 0.0286 | Yes |
| 3 | CENPF |  |  | 28 | 1.705 | 0.0399 | Yes |
| 4 | MYBL2 |  |  | 35 | 1.651 | 0.0521 | Yes |
| 5 | KNL1 |  |  | 38 | 1.629 | 0.0649 | Yes |
| 6 | E2F1 |  |  | 44 | 1.602 | 0.0769 | Yes |
| 7 | MKI67 |  |  | 45 | 1.598 | 0.0898 | Yes |
| 8 | MCM3 |  |  | 49 | 1.586 | 0.1021 | Yes |
| 9 | KIF15 |  |  | 52 | 1.568 | 0.1144 | Yes |
| 10 | PBK |  |  | 54 | 1.536 | 0.1267 | Yes |
| 11 | EZH2 |  |  | 55 | 1.534 | 0.1391 | Yes |
| 12 | GINS2 |  |  | 57 | 1.528 | 0.1513 | Yes |
| 13 | MEIS2 |  |  | 58 | 1.521 | 0.1636 | Yes |
| 14 | CDK1 |  |  | 69 | 1.488 | 0.1737 | Yes |
| 15 | SMC2 |  |  | 73 | 1.466 | 0.1850 | Yes |
| 16 | CHAF1A |  |  | 77 | 1.441 | 0.1961 | Yes |
| 17 | FBXO5 |  |  | 82 | 1.422 | 0.2068 | Yes |
| 18 | TACC3 |  |  | 116 | 1.334 | 0.2112 | Yes |
| 19 | NSD2 |  |  | 124 | 1.325 | 0.2206 | Yes |
| 20 | SMC4 |  |  | 127 | 1.320 | 0.2309 | Yes |
| 21 | BARD1 |  |  | 132 | 1.310 | 0.2407 | Yes |
| 22 | CCNF |  |  | 158 | 1.250 | 0.2460 | Yes |
| 23 | POLQ |  |  | 160 | 1.245 | 0.2559 | Yes |
| 24 | TMPO |  |  | 161 | 1.243 | 0.2659 | Yes |
| 25 | CDKN3 |  |  | 163 | 1.239 | 0.2758 | Yes |
| 26 | LMNB1 |  |  | 165 | 1.238 | 0.2856 | Yes |
| 27 | KIF23 |  |  | 167 | 1.235 | 0.2954 | Yes |
| 28 | NASP |  |  | 180 | 1.212 | 0.3029 | Yes |
| 29 | DBF4 |  |  | 189 | 1.197 | 0.3111 | Yes |
| 30 | KIF4A |  |  | 191 | 1.193 | 0.3205 | Yes |
| 31 | PRC1 |  |  | 211 | 1.161 | 0.3262 | Yes |
| 32 | KIF22 |  |  | 213 | 1.157 | 0.3354 | Yes |
| 33 | UBE2C |  |  | 214 | 1.154 | 0.3448 | Yes |
| 34 | CDKN2C |  |  | 223 | 1.143 | 0.3525 | Yes |
| 35 | CDC45 |  |  | 228 | 1.138 | 0.3609 | Yes |
| 36 | MCM2 |  |  | 238 | 1.130 | 0.3683 | Yes |
| 37 | NDC80 |  |  | 240 | 1.129 | 0.3773 | Yes |
| 38 | MCM5 |  |  | 243 | 1.127 | 0.3860 | Yes |
| 39 | TPX2 |  |  | 248 | 1.122 | 0.3943 | Yes |
| 40 | SMC1A |  |  | 251 | 1.120 | 0.4030 | Yes |
| 41 | RAD21 |  |  | 256 | 1.109 | 0.4112 | Yes |
| 42 | BRCA2 |  |  | 274 | 1.089 | 0.4167 | Yes |
| 43 | BUB1 |  |  | 277 | 1.084 | 0.4251 | Yes |
| 44 | MCM6 |  |  | 282 | 1.080 | 0.4331 | Yes |
| 45 | AURKB |  |  | 297 | 1.068 | 0.4390 | Yes |
| 46 | STMN1 |  |  | 307 | 1.062 | 0.4459 | Yes |
| 47 | CCNA2 |  |  | 309 | 1.057 | 0.4542 | Yes |
| 48 | CDC6 |  |  | 328 | 1.040 | 0.4592 | Yes |
| 49 | POLE |  |  | 344 | 1.023 | 0.4645 | Yes |
| 50 | EXO1 |  |  | 354 | 1.010 | 0.4710 | Yes |
| 51 | RBL1 |  |  | 358 | 0.999 | 0.4785 | Yes |
| 52 | CDKN1B |  |  | 360 | 0.997 | 0.4864 | Yes |
| 53 | KIF11 |  |  | 369 | 0.982 | 0.4928 | Yes |
| 54 | CASP8AP2 |  |  | 379 | 0.970 | 0.4989 | Yes |
| 55 | TTK |  |  | 383 | 0.966 | 0.5061 | Yes |
| 56 | MAD2L1 |  |  | 421 | 0.932 | 0.5065 | Yes |
| 57 | SAP30 |  |  | 424 | 0.930 | 0.5136 | Yes |
| 58 | KPNA2 |  |  | 435 | 0.919 | 0.5191 | Yes |
| 59 | CENPE |  |  | 437 | 0.918 | 0.5264 | Yes |
| 60 | LBR |  |  | 438 | 0.916 | 0.5338 | Yes |
| 61 | INCENP |  |  | 440 | 0.916 | 0.5410 | Yes |
| 62 | RACGAP1 |  |  | 443 | 0.912 | 0.5480 | Yes |
| 63 | ORC6 |  |  | 449 | 0.908 | 0.5544 | Yes |
| 64 | STIL |  |  | 455 | 0.903 | 0.5607 | Yes |
| 65 | HMGN2 |  |  | 466 | 0.900 | 0.5661 | Yes |
| 66 | FOXN3 |  |  | 491 | 0.876 | 0.5685 | Yes |
| 67 | UBE2S |  |  | 514 | 0.854 | 0.5712 | Yes |
| 68 | PLK1 |  |  | 526 | 0.850 | 0.5759 | Yes |
| 69 | BIRC5 |  |  | 541 | 0.842 | 0.5800 | Yes |
| 70 | CDC25B |  |  | 566 | 0.820 | 0.5820 | Yes |
| 71 | E2F2 |  |  | 576 | 0.814 | 0.5869 | Yes |
| 72 | MEIS1 |  |  | 588 | 0.807 | 0.5913 | Yes |
| 73 | TRA2B |  |  | 596 | 0.802 | 0.5964 | Yes |
| 74 | STAG1 |  |  | 602 | 0.800 | 0.6019 | Yes |
| 75 | HMMR |  |  | 622 | 0.788 | 0.6046 | Yes |
| 76 | CENPA |  |  | 627 | 0.786 | 0.6102 | Yes |
| 77 | CDC25A |  |  | 631 | 0.782 | 0.6159 | Yes |
| 78 | HNRNPD |  |  | 659 | 0.769 | 0.6169 | Yes |
| 79 | TFDP1 |  |  | 711 | 0.742 | 0.6131 | Yes |
| 80 | PLK4 |  |  | 720 | 0.739 | 0.6175 | Yes |
| 81 | CTCF |  |  | 729 | 0.731 | 0.6219 | Yes |
| 82 | AURKA |  |  | 761 | 0.718 | 0.6217 | Yes |
| 83 | CHEK1 |  |  | 779 | 0.709 | 0.6241 | Yes |
| 84 | RAD54L |  |  | 790 | 0.704 | 0.6279 | Yes |
| 85 | DTYMK |  |  | 800 | 0.700 | 0.6318 | Yes |
| 86 | NUP50 |  |  | 875 | 0.668 | 0.6229 | Yes |
| 87 | NEK2 |  |  | 901 | 0.659 | 0.6233 | Yes |
| 88 | KIF20B |  |  | 934 | 0.647 | 0.6224 | Yes |
| 89 | PTTG1 |  |  | 936 | 0.647 | 0.6274 | Yes |
| 90 | CKS2 |  |  | 966 | 0.634 | 0.6269 | Yes |
| 91 | PRIM2 |  |  | 968 | 0.633 | 0.6319 | Yes |
| 92 | SMARCC1 |  |  | 1049 | 0.608 | 0.6213 | No |
| 93 | CDC7 |  |  | 1060 | 0.604 | 0.6242 | No |
| 94 | SRSF2 |  |  | 1084 | 0.597 | 0.6246 | No |
| 95 | PDS5B |  |  | 1140 | 0.580 | 0.6186 | No |
| 96 | HMGB3 |  |  | 1154 | 0.577 | 0.6208 | No |
| 97 | POLA2 |  |  | 1161 | 0.574 | 0.6243 | No |
| 98 | E2F3 |  |  | 1223 | 0.555 | 0.6169 | No |
| 99 | SRSF10 |  |  | 1289 | 0.535 | 0.6087 | No |
| 100 | KIF2C |  |  | 1309 | 0.530 | 0.6093 | No |
| 101 | CCNB2 |  |  | 1373 | 0.513 | 0.6012 | No |
| 102 | TRAIP |  |  | 1402 | 0.504 | 0.5999 | No |
| 103 | RPS6KA5 |  |  | 1410 | 0.502 | 0.6026 | No |
| 104 | KMT5A |  |  | 1489 | 0.483 | 0.5914 | No |
| 105 | CKS1B |  |  | 1502 | 0.479 | 0.5929 | No |
| 106 | HNRNPU |  |  | 1548 | 0.470 | 0.5880 | No |
| 107 | TNPO2 |  |  | 1632 | 0.452 | 0.5756 | No |
| 108 | ILF3 |  |  | 1698 | 0.436 | 0.5665 | No |
| 109 | ESPL1 |  |  | 1733 | 0.430 | 0.5634 | No |
| 110 | NUMA1 |  |  | 1832 | 0.413 | 0.5477 | No |
| 111 | TENT4A |  |  | 2014 | 0.386 | 0.5157 | No |
| 112 | XPO1 |  |  | 2091 | 0.374 | 0.5040 | No |
| 113 | KATNA1 |  |  | 2424 | 0.332 | 0.4423 | No |
| 114 | RBM14 |  |  | 2453 | 0.327 | 0.4395 | No |
| 115 | SRSF1 |  |  | 2505 | 0.322 | 0.4323 | No |
| 116 | RPA2 |  |  | 2570 | 0.314 | 0.4224 | No |
| 117 | JPT1 |  |  | 2712 | 0.298 | 0.3975 | No |
| 118 | BUB3 |  |  | 2806 | 0.288 | 0.3817 | No |
| 119 | MTF2 |  |  | 2838 | 0.285 | 0.3780 | No |
| 120 | ODF2 |  |  | 2911 | 0.278 | 0.3663 | No |
| 121 | ARID4A |  |  | 3112 | 0.259 | 0.3296 | No |
| 122 | EWSR1 |  |  | 3146 | 0.256 | 0.3253 | No |
| 123 | PRMT5 |  |  | 3258 | -0.260 | 0.3059 | No |
| 124 | CUL4A |  |  | 3374 | -0.278 | 0.2858 | No |
| 125 | AMD1 |  |  | 3419 | -0.285 | 0.2796 | No |
| 126 | MAP3K20 |  |  | 3668 | -0.326 | 0.2341 | No |
| 127 | DR1 |  |  | 3888 | -0.365 | 0.1946 | No |
| 128 | SQLE |  |  | 4082 | -0.405 | 0.1605 | No |
| 129 | HIF1A |  |  | 4130 | -0.417 | 0.1547 | No |
| 130 | TOP1 |  |  | 4335 | -0.483 | 0.1191 | No |
| 131 | NOTCH2 |  |  | 4355 | -0.488 | 0.1193 | No |
| 132 | ATF5 |  |  | 4467 | -0.525 | 0.1021 | No |
| 133 | SLC7A5 |  |  | 4513 | -0.547 | 0.0978 | No |
| 134 | PML |  |  | 4765 | -0.689 | 0.0547 | No |
| 135 | SLC38A1 |  |  | 4773 | -0.694 | 0.0589 | No |
| 136 | MT2A |  |  | 4799 | -0.714 | 0.0599 | No |
| 137 | TGFB1 |  |  | 4944 | -0.861 | 0.0389 | No |
| 138 | HMGA1 |  |  | 4962 | -0.878 | 0.0427 | No |
| 139 | MYC |  |  | 5020 | -0.972 | 0.0395 | No |
| 140 | TLE3 |  |  | 5223 | -1.681 | 0.0140 | No |
Table: GSEA details [plain text format]

  

Fig 2: HALLMARK\_G2M\_CHECKPOINT: Random ES distribution      
 Gene set null distribution of ES for **HALLMARK\_G2M\_CHECKPOINT**
